# Small intestinal mucosa expression of putative chaperone fls485

**DOI:** 10.1186/1471-230X-10-27

**Published:** 2010-03-07

**Authors:** Andrea Reinartz, Josef Ehling, Susanne Franz, Verena Simon, Ignacio G Bravo, Claudia Tessmer, Hanswalter Zentgraf, Stefan Lyer, Ursula Schneider, Jan Köster, Kerstin Raupach, Elke Kämmerer, Christina Klaus, Jens JW Tischendorf, Jürgen Kopitz, Angel Alonso, Nikolaus Gassler

**Affiliations:** 1 Institute of Pathology, RWTH Aachen University, Aachen, Germany; 2 German Cancer Research Center, Heidelberg, Germany; 3 Department of Molecular Genome Analysis, German Cancer Research Center, Heidelberg, Germany; 4 Department of Pediatrics, RWTH Aachen University, Aachen, Germany; 5 Department of Medicine III, RWTH Aachen University, Aachen, Germany; 6 Institute of Pathology, University of Heidelberg, INF 220/221, 69120 Heidelberg, Germany

## Abstract

**Background:**

Maturation of enterocytes along the small intestinal crypt-villus axis is associated with significant changes in gene expression profiles. *fls485 *coding a putative chaperone protein has been recently suggested as a gene involved in this process. The aim of the present study was to analyze *fls48*5 expression in human small intestinal mucosa.

**Methods:**

*fls485 *expression in purified normal or intestinal mucosa affected with celiac disease was investigated with a molecular approach including qRT-PCR, Western blotting, and expression strategies. Molecular data were corroborated with several *in situ *techniques and usage of newly synthesized mouse monoclonal antibodies.

**Results:**

fls485 mRNA expression was preferentially found in enterocytes and chromaffine cells of human intestinal mucosa as well as in several cell lines including Rko, Lovo, and CaCo2 cells. Western blot analysis with our new anti-fls485 antibodies revealed at least two fls485 proteins. In a functional CaCo2 model, an increase in fls485 expression was paralleled by cellular maturation stage. Immunohistochemistry demonstrated fls485 as a cytosolic protein with a slightly increasing expression gradient along the crypt-villus axis which was impaired in celiac disease Marsh IIIa-c.

**Conclusions:**

Expression and synthesis of fls485 are found in surface lining epithelia of normal human intestinal mucosa and deriving epithelial cell lines. An interdependence of enterocyte differentiation along the crypt-villus axis and fls485 chaperone activity might be possible.

## Background

Sequential expression of genes and translation of the related molecules are generally assumed as a fundamental regulatory algorithm in development and cellular differentiation. In human small intestine, the crypt-villus axis (CVA) is one important example for cellular differentiation [1]. Epithelial cells migrate upward and downward the axis starting from the stem cell pools anchored adjacent to the crypt basis with a migration out of the crypt onto upper areas. Along the CVA, structural differentiation and functional specialization of enterocytes occur in a few days and are associated with a significant change in the panel of genes expressed [2]. This cellular differentiation is highly hampered in celiac disease, a disorder morphologically characterized by intraepithelial lymphocytosis, destruction of villi, and hyperplasia of crypts triggered by ingestion of gluten proteins contained in wheat, barley, and rye [3]. The spectrum of consecutive morphological changes in mucosal architecture of the small intestine is systematically addressed in the Marsh classification [4,5]. Evidence is given that gluten affects differentiation-associated genes in enterocytes [6], confers susceptibility to adenocarcinomas in human small intestine [7], and is associated with redox imbalance in intestinal mucosa and blood probably due to overproduction of free radicals [8,9].

Recently, an expression analysis of small intestinal enterocytes laser microdissected from the CVA was performed using Affymetrix X3P arrays containing 61,359 sequences representing approximately 47,000 transcripts [10]. In this setting, 415 genes were found predominantly expressed in the villus lining enterocytes and one of these was *fls485*. The gene *fls485 *(LOC51006; C3orf32), which was firstly identified in a cDNA library prepared from fetal liver mRNA (accession number: AB024705), maps to chromosome 3p25.3. LOC51066 (C3orf32) includes at least three open reading frames (ORF) which are assumed to encode various translation products probably with different functional relevance (for reference see relevant NCBI and EMBL data bases; accession numbers: Q9Y2M2, BAA76932, NM_015931, NP_057015). However, a translation product of about 39 kDa with wide tissue distribution including human small intestine is favored, but experimental evidence to verify existence of the protein is not given up to now.

Sequence analysis of the putative human fls485 protein revealed conserved DnaJ-class molecular chaperone domains [11]. In *Escherichia coli *DnaJ is a homodimeric molecule composed of four successive N-terminal regions representing functional domains: a J-domain (initial 73 amino acids of the *Escherichia coli *protein; HPD motif in loop regions), a glycine- and phenylalanine-rich G/F domain (residues 77-107), a central zinc-binding cysteine-rich CR-domain (residues 144-200), and a less conserved C-terminal domain [12,13]. DnaJ, a primary Hsp40 homologue, interacts specifically with DnaK, a Hsp70 protein, to participate in cellular processes like protein folding, transport, and degradation of misfolded proteins [14-16]. Sequence alignments of fls485 (NP_057015) revealed at least four zinc finger-like domain repeats of -CXXCXGXG-encoded by exons 4, 5, and 6. Exons 5 and 6 additionally encoded truncated motifs of -CXXCXG-. In general, -CXXC-sequences are assumed to be specific motifs for the thiol-/disulfide active sites of oxidoreductase members of the thioredoxin super-family [17]. *fls485 *is in discussion to be a candidate tumour suppressor gene, because it is mapped close to the uveal melanoma susceptibility locus *UVM2 *at 3p25 [18].

At present, fls485 protein synthesis is not shown in human tissues, and functional investigations concerning fls485 proteins are not published. The aim of the present study was to analyze expression of the *fls485 *gene and synthesis of respective proteins in human small intestinal mucosa of normal or disturbed CVA.

## Methods

### Tissues and cell culture

Normal small and large intestinal mucosa (n = 12; mean age, 68 years; range, 43 to 83 years; gender, m = 7, w = 5) mechanically dissected from the underlying tissues in surgical resections for sporadic cancer of the ascending colon were used for basic molecular and *in situ *analyses. Additionally, biopsies of normal small intestinal mucosa (n = 28; mean age, 40 years; range, 12 to 74 years; gender, m = 10, w = 18) as well as of small intestinal mucosa affected with celiac disease (n = 14; mean age, 58 years; range, 26 to 79 years; gender, m = 9, w = 5) were investigated. Tissues with celiac disease were designated as Marsh I (n = 2), Marsh II (n = 4), or Marsh III (total n = 8; IIIa, n = 2; IIIb, n = 4; IIIc, n = 2) according to Marsh-classification and criteria of the European Society of Pediatric Gastroenterology and Nutrition (ESPGAN) [4,5]. The use of human tissues was approved by each patient and the local ethics committee of the RWTH Aachen University. All diagnoses were established by conventional clinical and histological criteria [4,5].

### Generation of mouse monoclonal antibodies

The human fls485 sequence (bp 543 - 1017) was amplified by PCR as a *BamH*I/*Hind*III fragment (from the RZPD clone ID IRAUp969F10104D6) and cloned into the IPTG inducible expression vector pQE-8 (Qiagen, Hilden, Germany) resulting in a fusion protein with a C-terminal hexa-histidine tagg. The protein was purified using Ni-chelate chromatography (Qiagen). The urea fraction of the purified protein was used to immunize male mice employing standard procedures. The first injections of the protein were in Freund's complete adjuvant (Sigma, Deisenhofen, Germany). Subsequent injections were administered with antigen dissolved in PBS. Monoclonal antibodies were raised essential according to the method described [19]. Screening of the hybridomas for antibody production was performed using ELISA and immunoblot techniques following standard protocols [20].

### Cloning and expression of tagged fls485

A 474 bp fls485 fragment named fls485^158 ^was amplified from the pReceiver-MO2-fls485 distributed by the RZPD and cloned into *Hind*III - *Xho*I sides of pEGFP-C1 (Invitrogen) controlled by sequencing on both strands. Transient transfections were carried out in 3T3 cells with lipofectamine 2000 as recommended by the manufacturer (Invitrogen). Efficiency of transient transfections was evaluated with a Nikon fluorescence microscope and appropriate software (Nikon, Düsseldorf, Germany).

### Preparation of RNA and protein

Small or large intestinal mucosa specimens dissected from surgical resections or small intestinal mucosal biopsies were homogenized in TriReagent (Sigma). RNA and protein were simultaneously extracted according to Chomczynski's method [21]. Proteins were assayed by the BioRad approach (BioRad, München, Germany). Final protein preparations in Laemmli buffer were stored at -20°C until use.

### Reverse transcription and polymerase chain reaction

3 μg of DNase-digested total RNA was used for oligo (dT) primed first strand cDNA synthesis with SuperScript amplification (Invitrogen) following manufacturer's suggestions. Reverse transcription was followed by RNase H digestion step (20 min at 37°C). Control experiments included substitution of the enzyme reverse transcriptase by distilled water and transcription of a commercially provided RNA (50 ng). PCR analyses were performed in the LightCycler system (Roche-Diagnostics, Mannheim, Germany) following routine protocols [10]. Briefly, respective cDNAs were amplified using a well adapted set of primers corresponding to the coding sequences of human *fls485 *(primer pair I, exon 1 through exon 3: 5'-GAG ACC TCG TCA TCC AGG AG-3', 5'-TTG ACC AGT GAC GAG TGA GG-3'; primer pair II, exon 8 through exon 9: 5'-TGC AGC AGC GCC AGA C-3', 5'-ACA GCA ATA CCG CTC AGG AT-3'). PCR was performed in 20 μl aliquots consisting of 2 mM MgCl_2_, 0.5 pM each primer, 2 μl SYBR green mix, and 2 μl cDNA [45 × (95°C; 10 s/60°C; 15 s/72°C; 10 s)]. For amplification of villin transcripts the following set of primers were used: 5'-AGC TTA TCA AGC CGT CAT CC-3', 5'-CCC GGT CTC CAA GTT GTT AG-3'. The PCR conditions were identical to fls485 PCRs. *Amplicon *integrity was evaluated by fluorescence reading, melting step, and 2% agarose ethidium bromide gels. Quantification of mRNA expression levels between groups was performed when probes were normalized to the GAPDH content. Differences between groups were evaluated with the two-paired t-test.

### mRNA in situ hybridization

A fragment of human fls485 (NM_015931; 544-1017) was amplified by RT-PCR and cloned into pBluescript II KS (-) (Invitrogen, Karlsruhe, Germany). Orientation of the inserts was determined by sequencing. Plasmids were restricted with *Bam*H1 or *Hind*III and then transcribed with T3 (antisense) or T7 (sense) RNA polymerase. Dewaxed small intestinal tissue sections were proteinase K treated (Roche-Diagnostics; 8 μg/ml in PBS; 30 min at 37°C), acetylated, and then incubated with Dig-labeled riboprobes (7 or 14 ng/μl) at 47°C overnight. AP-labeled anti-Dig antibodies and appropriate chromogens were applied for visualization.

### Western blot analysis

Proteins in Laemmli sample buffer were resolved by SDS-PAGE (10%) and transferred to PVDF Immobilon-P membrane. For molecular weight estimation of proteins, Rainbow molecular weight marker was used following manufacturer's suggestions (Amersham Pharmacia Biotech, Little Chalfont, England). The following antibodies were used: mouse anti-human beta-actin (0.4 μg/ml; Santa Cruz Biotechnology, Santa Cruz, USA), mouse anti-human fls485 (clone #7 or clone #10 and subclones), and secondary HRP-conjugated antibodies (1:10,000; Santa Cruz Biotechnology, Santa Cruz, USA). His- or EGFP-taggs were detected with antibodies directed against 6xHis (R&D Systems, Wiesbaden, Germany) or EGFP (OpenBiosystems, Huntsville, USA). The ECL substrate (Amersham) was applied following manufacturer's recommendations. Negative controls included blots in which the primary antibody was omitted.

### Immunohistochemistry

Sections of paraffin-embedded small intestinal tissues or cryo-conserved specimens were used following routine protocols [10]. Briefly, paraffin-embedded sections were dewaxed and incubated in citrate buffer pH 6.0 for 30 min at 95°C. The cryo-sections were fixed with acetone for 10 min at -20°C. Afterwards sections were washed in PBS, treated with 1% normal serum, and then incubated with the primary anti-fls485 antibody for 1 h at room temperature in a moist chamber. Sections were then washed in PBS and incubated for 30 min with the secondary biotinylated goat anti-mouse IgA antibody (DAKO, Glostrup, Denmark) diluted 1:200 in PBS. The ABC detection kit (VECTOR, Burlingame, USA) and DAB (DAKO) as well as Cy2-, Cy3- (both Dianova, Hamburg, Germany) or FITC-fluorochromes (Jackson ImmunoResearch, Baltimore, USA) were used as suggested by the providers. In negative controls sections were similarly processed but the appropriate normal serum was used or the primary antibody had been totally omitted. For semi-quantitative evaluation of immunostainings a value representing the product of staining intensity (1 through 3) and the number of specifically immunostained cells (0 = negative, 1 = < 10%, 2 = 10 - 50%, 3 = 51 - 80%, 4 = > 80%) were used. Differences between groups were evaluated with two-paired t-test.

### Cell culture and transfection

For cell culture experiments Rko cells (ATCC: CRL-2577), Lovo cells (ATCC: CCL-229), and CaCo2 cells (ATCC: HTB-37), all human colon cancer cell lines, or 3T3 fibroblasts (ATCC: CCL-92) were used. All cells were maintained in 5% CO_2 _atmosphere at 37°C. Rko cells were maintained in culture with RPMI1640, whereas DMEM with 2 mM L-glutamine was used for Lovo and 3T3 cells. CaCo2 cells were cultured in EMEM supplemented with 2 mM L-glutamine. Media were supplemented with 5 - 10% fetal bovine serum. All transfections were performed with lipofectamine 2000 (Invitrogen) following manufacturer's recommendations. For functional experiments, CaCo2 cells were seeded with a cell number of 10^4^/cm^2 ^(group I: low cell number) or 10^5^/cm^2 ^(group II: high cell number). Cell growth and maturation were controlled by microscopy and villin mRNA expression. Villin gene expression has been demonstrated as valuable biomarker for differentiation of enterocytes and CaCo2 cells [22-24]. Confluence of cells was found in group II cells after a time period of three days. All cells were harvested, total RNA was prepared, and fls485 as well as villin RT-PCR were performed as described above. The results from three separate experiments were used for statistical analysis.

## Results

### Establishment and characterization of monoclonal antibodies recognizing fls485

**Figure 1:**
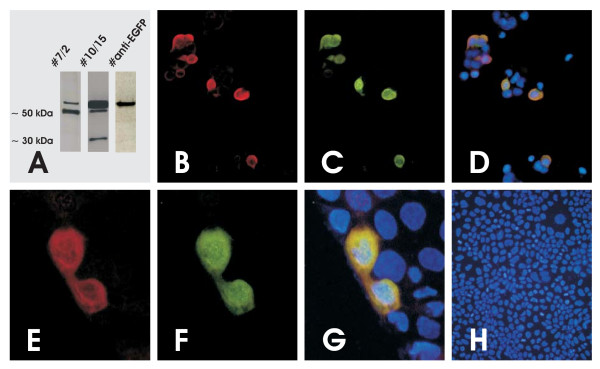
**Characterization of anti-fls485 antibodies**. Detection of chimeric EGFP-fls485^158 ^protein in transfected 3T3 (A: Western blot) or CaCo2 cells (B-H: immunofluorescence) with different anti-fls485 antibodies. (A) Western blots of EGFP-fls485^158 ^expressed in 3T3 cells incubated with antibodies directed against fls485^158 ^clone/subclone #7 (left) or clone/subclone #10 (middle). Endogenous fls485 expression by 3T3 cells is detectable as a signal about 55 kDa when incubated with the anti-fls485 clones. One additional signal about 35 kDa is exclusively found when clone/subclone #10 is used. EGFP-fls485^158 ^expression in 3T3 transfectants was additionally visualized with anti-EGFP antibodies as control (right). (B-D) Immunofluorescence of EGFP-fls485^158 ^protein in transfected CaCo2 cells incubated with clone/subclone #7, secondary antibody Cy3-labeled (B), anti-EGFP, secondary antibody FITC-labeled (C), and overlay (D). (E-G) Immunofluorescence of EGFP-fls485^158 ^protein in transfected CaCo2 cells incubated with clone/subclone #10, secondary antibody Cy3-labeled (E), anti-EGFP, secondary antibody FITC-labeled (F), and overlay (G). (H) Negative control; EGFP-fls485^158 ^transfected CaCo2 cells after incubation with all secondary antibodies.

Immunization of mice, cellular fusion, and subsequent purification revealed a panel of IgG ELISA positive mAbs recognizing epitopes within the target-peptide of fls485. Positive clones #7 and #10 were ELISA-detected and a panel of subclones #7/1, #7/2, and #10/15 was established. Specificity against fls485 was corroborated by expression of the fusion protein EGFP-fls485^158 ^in 3T3 cells. Western blotting of 3T3 transient EGFP-fls485^158 ^transfectants and probing of membranes with #7 or #10 clones/subclones revealed the fusion protein (approx. 61 kDa) which was additionally recognized with antibodies against the EGFP tagg (Figure 1A). The data were further substantiated by fluorescence imaging of EGFP and fls485/fls485^158 ^in CaCo2 transient transfectants (Figures 1B-H).

### fls485 mRNA and proteins are found in intestinal mucosa

**Figure 2:**
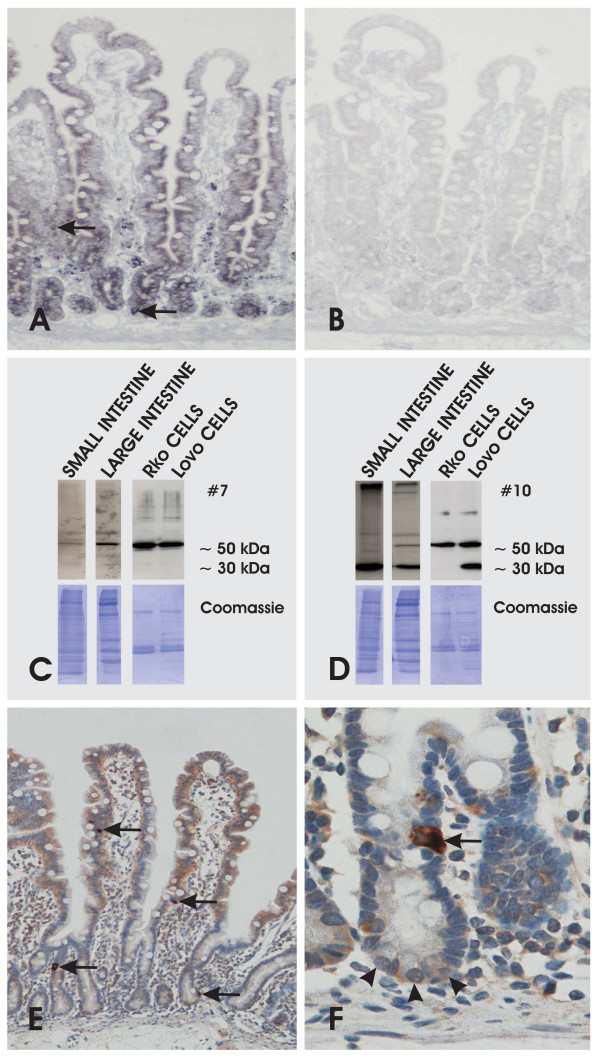
**Characterization of fls485 expression in intestinal mucosa**. fls485 expression in human intestinal mucosa with mRNA *in situ *hybridization (A and B), Western blot analysis (C and D), or immunohistochemistry (E and F). (A) Distribution of fls485 mRNA in human small intestinal mucosa (antisense riboprobe). The arrows mark strong mRNA accumulation in putative chromaffin cells (original magnification approx. × 200). (B) Serial section of (A) incubated with fls485 sense riboprobe (original magnification approx. × 200). (C) Blotted protein lysates from human small and large intestinal mucosa as well as Rko and Lovo cells after incubation with clone #7. (D) Blotted lysates as shown in (C) probed with clone #10. (E) fls485 protein is found with an increasing gradient along the CVA (clone #10) (original magnification approx. × 200). Chromaffin cells are marked by arrows. Note some interstitial cells are stained. (F) Strong fls485 immunostaining is found in a chromaffin cell (arrow), whereas Paneth cells (arrowheads) are only slightly or not stained (clone #10) (original magnification approx. × 400).

In RT-PCRs, fls485 mRNA was detectable with primer pair I and II in all normal small intestinal mucosa probes investigated (n = 31; data not shown). These data were substantiated with mRNA *in situ *hybridization showing enterocytes and chromaffine cells as important sources of fls485 mRNA (Figure 2A,B). Along the CVA, fls485 mRNA was preferentially detectable in lower parts. An increasing mRNA gradient as suggested by previous work [10] was not detectable. Additional fls485 mRNA signaling was found in interstitial cells, probably lymphocytes.

In order to investigate the fls485 protein pattern, Western blot analysis of human small and large intestinal mucosa as well as of two intestinal cell lines, Rko and Lovo, was performed. PVDF membranes were probed with the newly established anti-fls485 antibodies (Figure 2C,D). The different subclones of #7 and #10 founders regularly revealed a signal about 55 kDa. In addition, a second strong signal (approx. 35 kDa) was found with clone #10 and subclone #10/15 in preparations of small intestinal mucosa and Lovo, but not in Rko cells. However, the 35 kDa signal was not detectable with #7 clones. Interestingly, a difference in the distribution of the two protein signals was revealed when comparing small and large intestinal mucosa. The 55 kDa molecule was preferentially found in preparations from large intestinal mucosa, whereas the small intestinal mucosa was dominated by the protein with lower molecular weight. In addition, the protein distribution pattern of large intestinal mucosa was similar to the findings with Lovo and Rko cells (Figure 2C,D)

Immunostaining against fls485 proteins with #7 or #10 clones/subclones revealed protein localization in the surface lining epithelium occasionally with a slightly increasing gradient from crypts to villi (Figure 2E). In enterocytes from small and large intestine, the protein displayed a constant cytoplasmic distribution without any visible co-localization to cellular membranes. The surface-lining enterocytes were intermingled with some cells strongly immunostained for fls485 protein and morphologically characterized by an apical nucleus and baso-lateral cytoplasm with infranuclear granules characteristic for chromaffin cells (Figure 2F). Anti-fls485 immunostaining of Paneth cells was very weak or not detectable, whereas some lamina propria cells preferential lymphocytes were positive. Results from immunostainings did not differ between #7 and #10 subclones.

### Impaired expression of fls485 in celiac disease

**Figure 3:**
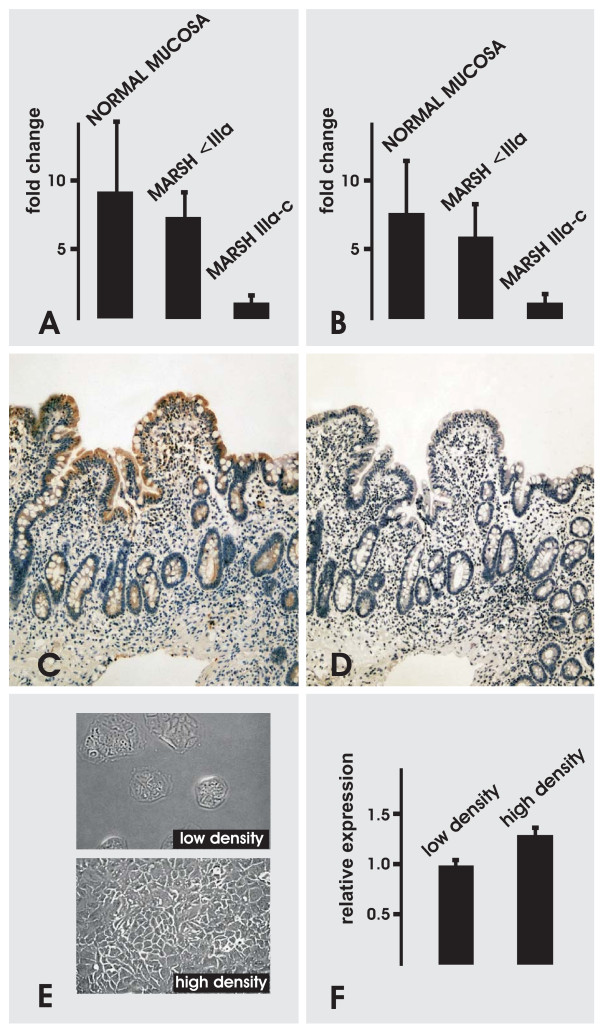
**Impaired fls485 expression in celiac disease**. fls485 expression is impaired in human small intestinal mucosa affected with celiac disease classified Marsh IIIa-c when compared with normal mucosal specimens (controls). (A) fls485 mRNA expression in small intestinal mucosa detected with primer pair I. (B) fls485 mRNA expression detected with primer pair II in the identical probes as shown in (A). (C) Anti-fls485 immunohistochemistry (clone #7/2) of intestinal mucosa classified Marsh IIIb (original magnification approx. × 200). (D) Serial tissue section of (C) incubated with normal serum as negative control (original magnification approx. × 200). (E) Morphological aspects of CaCo2 cells cultured with low or high cellular density (original magnification approx. × 200). (F) fls485 expression normalized to GAPDH is increased in high density CaCo2 cell cultures (primer pair I, *p *< 0.05).

In order to prove a putative link between CVA behavior and fls485 expression, small intestinal mucosa affected with CVA disturbing celiac disease was investigated. Using qRT-PCR (primer pair I and II) fls485 mRNA expression was significantly decreased in mucosal biopsies affected with celiac disease Marsh IIIa-c (n = 8) when compared with normal controls (n = 28) (primer pair I: 7.2 fold, *p *< 0.0001; primer pair II: 8.7 fold, *p *< 0.0001). In celiac diseased biopsies smaller than Marsh IIIa stages (n = 6) fls485 mRNA levels were similar to controls (primer pair I: 1.3 fold, *p *> 0.05; primer pair II: 1.3 fold, *p *> 0.05) (Figure 3A,B). RT-PCR findings were in line with anti-fls485 immunostainings of celiac diseased tissues (Figure 3C,D) when compared with normal mucosa (Figure 2E). Diminished fls485 immunostaining with clone #7 and #10 or appropriate subclones was found in small intestinal biopsies with different stages of villus atrophy (Marsh IIIa-c). In such specimens, a compensatory fls485 protein increase in epithelia lining elongated crypts was never visible. However, the differences in fls485 tissue staining were not significant using a semi-quantitative scoring system with fls485 staining intensity and number of anti-fls485-stained cells as variables (*p *> 0.05).

The role of fls485 in cellular maturation was further addressed in a functional CaCo2 assay. Low and high density cultures of CaCo2 cells have been established as a well accepted model of cellular maturation reflecting the molecular events along the CVA [24]. In order to investigate fls485 expression in different stages of cellular maturation, CaCo2 cells were cultured in low or high density. In this experimental setting maturation of CaCo2 cells was paralleled by an increase of fls485 mRNA (primer pair I, *p *< 0.05) (Figure 3E,F).

## Discussion

Proliferation and differentiation of epithelial cells are generally associated with complex changes in gene expression and protein synthesis. Investigation of the molecular mechanisms involved is facilitated by descriptive studies of high-turnover epithelial differentiation as found in the CVA of human small intestinal mucosa. As recently shown, maturation of human enterocytes along the CVA is associated with differential expression of at least 778 genes including *fls485 *[10]. The gene *fls485 *(LOC51066) includes at least three ORFs and is suggested to encode a putative chaperon protein of approx. 39 kDa (accession numbers: Q9Y2M2, BAA76932) of unknown intestinal tissue distribution and function. We aimed to analyze distribution of fls485 mRNA and protein in enterocytes along the CVA of normal intestinal mucosa and in celiac disease which is characterized by sequential CVA destruction. In order to elucidate *fls485 *expression, fls485 tissue distribution, and putative functional impacts a panel of monoclonal antibodies was generated in mice using a fls485 core peptide. In a primary setting of our mice immunization experiments, two epitopes included in the fls485^49-206 ^sequence (EKKLLHFIQLV and KRKAKQSRR), both predicted to bind MHC molecules with high affinity, were separately applied. Using these peptides in mice, however, the experiments failed to generate useful monoclonal antibodies. However, in a second approach, powerful antibodies were established by using the fls485 core peptide. The so generated anti-fls485 antibodies provided different application options, including immunostaining and Western blotting. The disadvantage of this procedure, the unknown protein binding site of the antibodies, was compensated by extensive antibody characterization, comparison of staining results, and fls485 expression profiling as subsequently detailed.

Characterization of selected clones #7, #10, and dependent subclones was performed with a panel of well-adapted techniques. Firstly, ELISA screening against KLH or BSA identified founder clones #7 and #10. Secondly, transient transfections of 3T3 fibroblasts with a EGFP-fls485 construct resulted in expression of the EGFP-fls485^158 ^fusion protein, which was characterized with Western blotting and detection of the EGFP-tagg (anti-EGFP antibodies) or the fls485^158 ^protein (anti-fls485 antibodies #7 and #10 as well as subclones). Thirdly, co-localization of EGFP and fls485 immunosignaling was found in EGFP-fls485^158 ^3T3 and CaCo2 transfectants. Fourthly, immunostainings of sectioned paraffin-embedded normal human small intestinal mucosa with #7, #10 or subclones revealed an overlap with the fls485 mRNA *in situ *hybridization pattern. Fifthly, anti-fls485 immunostaining was constantly found intracytoplasmic which confirms the assumption of fls485 being a soluble intracytoplasmic molecule as predicted by its amino acid sequence. Sixthly, corresponding to three ORFs of the *fls485 *gene and putative posttranslational protein modifications a protein of the predicted weight (approx. 35 kDa) and at least one additional protein (approx. 55 kDa) were repeatedly found in Western blotting. Our attempts to further purify the proteins from small intestinal enterocytes or cell lines were hampered by technical obstacles probably due to posttranslational protein modifications. In continuation of these experiments, we will establish stable fls485 transfectants and synthesize recombinant fls485 protein, which are both of high interest in biochemical and prospective functional fls485 analysis.

Immunostainings of fls485 protein revealed an increasing gradient from crypts to villi as anticipated from a previous mRNA expression study [10]. Using anti-fls485 mRNA *in situ *hybridization, the increasing fls485 mRNA gradient was not reproducible. We speculate that the phenomenon could be due to existence of different fls485 mRNA species which are incompletely hybridized with the riboprobes in use. However, current riboprobes included a putative overlapping sequence of respective mRNA species deriving from the three fls485 ORFs. In order to clarify this issue a detailed fls485 mRNA expression study using laser microdissected enterocytes from different CVA locations and mRNA *in situ *hybridization experiments with additional riboprobes is conceived. Another possibility for aberrant fls485 mRNA expression along the CVA could be a post-transcriptional control of fls485 protein synthesis. However, at present we have no data to further substantiate this assumption. As a final point it has to be noted here is that asymmetric distribution of mRNA and protein species along the CVA is possible and has been already demonstrated for other genes [23].

Our study gives evidence that fls485 mRNA and proteins are physiologically found in human intestinal mucosa as well as in several cell lines (including Lovo, Rko, CaCo2, HeLa, 3T3, HCT116, Capan1). However, at present there are no data available concerning the functional relevance of the fls485 protein and its different isoforms. We do hypothesize from the fls485 amino acid sequence consisting of at least four zinc finger-like domain repeats of -CXXCXGXG- (NP_057015) that fls485 could be a chaperone with thiol-disulfide oxidoreductase activity. Such motifs are additionally found in redox-regulated molecular chaperones such as Hsp33 [12,25], a protective molecule which might be directly involved in bacterial colonization of the intestine [26]. Bacterial overgrowth [27] and increased oxidative stress [28] are frequently found in patients suffering from celiac disease. It could be hypothesized that an impaired expression of the putative chaperone fls485 might be involved in the pathogenesis of such complications. Indeed, our present studies revealed impaired fls485 expression in human small intestinal mucosa affected with celiac disease Marsh IIIa-c, whereas no significant difference was detectable between diseased mucosal specimens classified lower than Marsh IIIa and normal intestinal mucosa (controls). This observation and our functional findings from CaCo2 cells argue for an interdependence of enterocyte differentiation along the CVA and *fls485 *expression. As stated above, the use of fls485 transfectants and recombinant fls485 protein as well as mucosal tissue cultures is suggested as promising functional approach to further investigate the phenomena.

## Conclusions

Here we show experimental evidence for fls485 mRNA expression and protein synthesis in human intestinal mucosa and impaired expression in celiac disease. We assume from *fls485 *gene structure, amino acid motifs, and Western blot analysis that at least two fls485 proteins might exist which could probably be involved in differentiation of the CVA. Our findings provide a framework for guiding further experiments to clarify consequences of aberrant fls485 expression in celiac-diseased intestinal tissues.

## Abbreviations used

CVA: crypt-villus axis

## Competing interests

The authors declare that they have no competing interests.

## Authors' contributions

AR and JE participated equally in characterization of monoclonal antibodies established by SF, CT, and HZ. Immunostainings were performed by VS, US, JK, and KR. IGB and SL performed cloning experiments. Clinical data were provided by EK and JJWT. Functional experiments were performed by US and CK. JK, AA, and NG participated in study design and coordination. All authors read and approved the final manuscript version.

## Pre-publication history

The pre-publication history for this paper can be accessed here:

http://www.biomedcentral.com/1471-230X/10/27/prepub

## References

[B1] Parnis S Nicoletti C Ollendorff V Massey-Harroche D Enterocytin: A new specific enterocyte marker bearing a B30.2-like domain J Cell Physiol 2004 198 441 451 10.1002/jcp.10418 14755549

[B2] Traber PG Silberg DG Intestine-specific gene transcription Annu Rev Physiol 1996 58 275 297 10.1146/annurev.ph.58.030196.001423 8815796

[B3] Schuppan D Hahn EG Biomedicine. Gluten and the gut-lessons for immune regulation Science 2002 297 2218 2220 10.1126/science.1077572 12351776

[B4] Marsh MN The natural history of gluten sensitivity: defining, refining and redefining QJM 1995 88 9 13 7894995

[B5] Oberhuber G Caspary WF Kirchner T Borchard F Stolte M Empfehlungen zur Zöliakie-/Spruediagnostik Pathologe 2001 22 72 81 10.1007/s002920000428 11225448

[B6] Juuti-Uusitalo K Mäki M Kainulainen H Isola J Kaukinen K Gluten affects epithelial differentiation-associated genes in small intestinal mucosa of coeliac patients Clin Exp Immunol 2007 150 294 305 17888028 10.1111/j.1365-2249.2007.03500.x PMC2219351

[B7] Howdle PD Jalal PK Holmes GK Houlston RS Primary small-bowel malignancy in the UK and its association with coeliac disease Q J Med 2003 96 345 353 10.1093/qjmed/hcg058 12702783

[B8] Rivabene R Mancini E De Vincenzi M In vitro cytotoxic effect of wheat gliadin-derived peptides on the Caco-2 intestinal cell line is associated with intracellular oxidative imbalance: implications for coeliac disease Biochim Biophys Acta 1999 1453 152 160 9989255 10.1016/s0925-4439(98)00095-7

[B9] Odetti P Valentini S Aragno I Garibaldi S Pronzato MA Rolandi E Barreca T Oxidative stress in subjects affected by celiac disease Free Radic Res 1998 29 17 24 10.1080/10715769800300031 9733018

[B10] Gassler N Newrzella D Böhm C Lyer S Li L Sorgenfrei O van Laer L Sido B Mollenhauer J Poustka A Schirmacher P Gretz N Molecular characterisation of non-absorptive and absorptive enterocytes in human small intestine Gut 2006 55 1084 1089 10.1136/gut.2005.073262 16556670 PMC1856251

[B11] Marchler-Bauer A Anderson JB Cherukuri PF DeWeese-Scott C Geer LY Gwadz M He S Hurwitz DI Jackson JD Ke Z Lanczycki CJ Liebert CA Liu C Lu F Marchler GH Mullokandov M Shoemaker BA Simonyan V Song JS Thiessen PA Yamashita RA Yin JJ Zhang D Bryant SH CDD: a conserved domain database for protein classification Nucleic Acids Res 2005 33 D192 196 10.1093/nar/gki069 15608175 PMC540023

[B12] Linke K Wolfram T Bussemer J Jakob U The roles of the two zinc binding sites in DnaJ J Biol Chem 2003 278 44457 44466 10.1074/jbc.M307491200 12941935

[B13] Shi YY Tang W Hao SF Wang CC Contributions of cysteine residues in Zn2 to zinc fingers and thiol-disulfide oxidoreductase activities of chaperone DnaJ Biochemistry 2005 44 1683 1689 10.1021/bi0480943 15683252

[B14] Farinha CM Nogueira P Mendes F Penque D Amaral MD The human DnaJ homologue (Hdj)-1/heat-shock protein (Hsp) 40 co-chaperone is required for the in vivo stabilization of the cystic fibrosis transmembrane conductance regulator by Hsp70 Biochem J 2002 366 797 806 12069690 10.1042/BJ20011717 PMC1222832

[B15] Hennessy F Cheetham ME Dirr HW Blatch GL Analysis of the levels of conservation of the J domain among the various types of DnaJ-like proteins Cell Stress Chap 2000 5 347 358 10.1379/1466-1268(2000)005<0347:AOTLOC>2.0.CO;2 PMC312864 11048657

[B16] Kelley WL The J-domain family and the recruitment of chaperone power TIBS 1998 23 222 227 9644977 10.1016/s0968-0004(98)01215-8

[B17] Raina S Missiakas D Making and breaking disulfide bonds Annu Rev Microbiol 1997 51 179 202 10.1146/annurev.micro.51.1.179 9343348

[B18] Tschentscher F Hüsing J Hölter T Kruse E Dresen IG Jöckel KH Anastassiou G Schilling H Bornfeld N Horsthemke B Lohmann DR Zeschnigk M Tumor classification based on gene expression profiling shows that uveal melanomas with and without monosomy 3 represent two distinct entities Cancer Res 2003 63 2578 2584 12750282

[B19] Köhler G Milstein C Continuous cultures of fused cells secreting antibody of predefined specificity Nature 1975 256 495 497 10.1038/256495a0 1172191

[B20] Zentgraf H Frey M Schwinn S Tessmer C Willemann B Samstag Y Velhagen I Detection of histidine-tagged fusion proteins by using a high-specific mouse monoclonal anti-histidine tag antibody Nucl Acids Res 1995 23 3347 3348 10.1093/nar/23.16.3347 7667114 PMC307199

[B21] Chomczynski P A reagent for the single-step simultaneous isolation of RNA, DNA and proteins from cell and tissue samples Biotechniques 1993 15 532 537 7692896

[B22] Dudouet B Robine S Huet C Sahuquillo-Merino C Blair L Coudrier E Louvard D Changes in villin synthesis and subcellular distribution during intestinal differentiation of HT29-18 clones J Cell Biol 1987 105 359 369 10.1083/jcb.105.1.359 2440895 PMC2114929

[B23] Landry C Huet C Mangeat P Sahuquet A Louvard D Crine P Comparative analysis if neutral endopeptidase (NEP) and villin gene expression during mouse embryogenesis and enterocyte maturation Differentiation 1994 56 55 65 8026647 10.1046/j.1432-0436.1994.56120055.x

[B24] Sambuy Y De Angelis I Ranaldi G Scarino ML Stammati A Zucco F The Caco-2 cell line as a model of the intestinal barrier: influence of cell and culture-related factors on Caco-2 cell functional characteristics Cell Biol Toxicol 2005 21 1 26 10.1007/s10565-005-0085-6 15868485

[B25] Graf PC Jakob U Redox-regulated molecular chaperones Cell Mol Life Sci 2002 59 1624 1631 10.1007/PL00012489 12475172 PMC11337458

[B26] Kumsta C Jakob U Redox-regulated chaperones Biochemistry 2009 48 4666 4676 10.1021/bi9003556 19368357 PMC2848813

[B27] Rana SV Bhardwaj SB Small intestinal bacterial overgrowth Scand J Gastroenterol 2008 43 1030 1037 10.1080/00365520801947074 18609165

[B28] Stojiljković V Todorović A Radlović N Pejić S Mladenović M Kasapović J Pajović SB Antioxidant enzymes, glutathione and lipid peroxidation in peripheral blood of children affected by coeliac disease Ann Clin Biochem 2007 44 537 543 10.1258/000456307782268075 17961308

